# Relationship between BI-RADS and the Results of the Wire-Guided Percutaneous Localization for Non-Palpable Breast Lesions

**DOI:** 10.51894/001c.9061

**Published:** 2019-07-01

**Authors:** Mohamad Dughayli, Jason DeFatta, Ayda Dashtaei, Amber Peace, Fadi Baidoun, Gregory Olson

**Affiliations:** 1 General Surgery Henry Ford Wyandotte Hospital; 2 Radiology Henry Ford Wyandotte Hospital

**Keywords:** bi-rads categorization, breast cancer, non-palpable breast lesions, needle localization

## Abstract

**INTRODUCTION:**

The aim of this study is to evaluate the relationship between Breast Imaging Reporting and Data System and surgical biopsies that may increase effectiveness of wire-guided percutaneous localizations for non-palpable breast lesions.

**METHODS:**

A retrospective review of a sample of 149 patients who underwent wire-guided localization with wide local excision for non-palpable breast lesions at the authors’ institution between January 2013 and April 2016. After IRB approval, sample patients’ records were reviewed and data were collected concerning their radiological, histological and surgical characteristics.

**RESULTS:**

One (0.67%) complication occurred related to wire migration. There were nine (6.04%) recorded cases of seroma and three (2.01%) cases of hematoma. Breast Imaging Reporting and Data System (BI-RADS) Category 4 was found to have a positive predictive value of 28.4% for breast cancer. Under Category 4 subcategorization 4A, 4B, and 4C, the number of positive lesions were two (6.89%), three (10.34%) and five (17.24%), respectively. Forty (78.43%) of the 51 patients with cancer had negative (i.e., non-cancerous) margins compared to 11 (21.57%) cases that had positive margins after the first procedure.

**CONCLUSION:**

The BI-RADS Category 4 encompasses the majority of lesions, with approximately 70% of such biopsies lesions later found to be benign. A subcategorization of BI-RADS 4 needs further clarification in distinguishing benign vs malignant imaging characteristics. Future retrospective studies designed to identify benign vs. malignant lesions, confirmed by validating prospective studies, will better clarify a new subcategorization of BI-RADS Category 4, thus allowing surgeons and radiologists to make the best surgical recommendations for their patients.

## INTRODUCTION

The extensive use of screening mammography has resulted in an increasing number of non-palpable malignant breast tumors being detected at an early stage.^1^ In most early stage breast cancers, radical mastectomy has been replaced by breast-conserving surgeries. This technique allows for the precise surgical removal of the tumor, and is cosmetically more acceptable to patients.^1^ At present, two techniques are widely used for the localization and removal of non-palpable breast tumors: Wire-guided localization (WGL)^2^ and radio-guided occult lesion localization (ROLL).^3,4^ WGL breast biopsy has shifted from a diagnostic to a therapeutic approach for more conservative treatments of non-palpable breast lesions. This technique, which has been used for decades, was first described by Kopans and DeLuca in 1980.^2^

The ROLL technique has been widely used in clinical practice since 1996. The ROLL method has recently been preferred by clinicians to the WGL method in the surgical treatment of many non-palpable breast lesions.^2^ WGL is still widely used as a therapeutic technique to facilitate more conservative approach in numerous institutions and remains the method utilized in the authors’ healthcare system.

There are three main goals for the surgical excision of non-palpable breast lesions. These include a) using an efficient and accurate preoperative localization technique to facilitate the complete excision of the lesion, b) total resection margin negativity (i.e., removal of all cancerous tissue), and c) avoiding excessive removal of healthy tissue. A few studies have suggested that the use of the WGL method results in a relatively high rate of post-resection positive (i.e., cancerous) margins, resulting in an increased risk of local recurrence and higher reoperation rates.^5,6^

When performing lumpectomy for breast cancer, obtaining a clear surgical margin is recognized as the strongest predictor of local control and may decrease recurrence rates by as much as 15%.^7^ Re-excision in these patients with a positive margin brings the risk of recurrence in line with those with initial clear margins. With this in mind, a resection margin of 1 mm or more is recommended.^8^ With the reported margin rates of WGL between 21%-43%,^9^ this technique remains a commonly used localization method and is used as the “gold-standard” among newer diagnostic techniques.^10,11^

The Breast Imaging Reporting and Data System Classification (BI-RADS) is an accepted radiological risk and quality assessment tool proposed by the American College of Radiology to clarify breast lesions into six categories labeled 0 to 6.^12^ With this tool, 0 is incomplete, 1 is negative, 2 is benign, 3 is probably benign, 4 is suspicious, 5 is highly suspicious and 6 is a known biopsy proven malignancy.^12^

### Purpose of Study

The aims of this study were to: a) evaluate the efficacy of the BI-RADS classification system with WGL excisions performed for non-palpable breast lesions with suspicious findings on imaging, and b) evaluate the most recent 2006 BI-RADS protocol to classify higher-category breast lesion patients.

## METHODS

After IRB approval, the electronic health records of patients who underwent WGL at our institution between January 2013 and April 2016 for non-palpable breast radiological lesion abnormalities were reviewed and pertinent data collected. We included all patients who underwent breast conservative surgery for non-palpable tumors. Those patients with palpable tumors, mastectomy or pregnancy were excluded from the study.

The hospital electronic health record system was searched for relevant  radiological, surgical, pathological, and clinical characteristics. Patient characteristics collected included age, location of abnormal radiological lesion, radiological appearance, BI-RADS assessment category, size on imaging, localization method, postoperative histopathological findings (i.e., tumor type), histopathological grade, histopathological size, and margin status (positive or negative).

Patients suitable for study inclusion were defined as those having undergone lumpectomy (wide local excision) for non-palpable, possibly malignant breast lesions. Patients with lesions suggesting malignancy (BI-RADS Categories 4 and 5) on mammography/ultrasound and those with benign-appearing, solid nodules (BI-RADS 3 on mammogram/ultrasound), as well as female patients with dense radiological findings and a strong family history (“strong” defined in this study as multiple family member, e.g., a 1^st^ and 2^nd^ degree relative, multiple 1^st^ degree relatives, or multiple 2^nd^ degree relatives) of breast cancer were also included.

The breast needle localization protocol at our institution was as follows. Appropriate prior breast imaging was first obtained and reviewed. If there had been prior breast ultrasound and the lesion was easily visualized by sonography, needle localization by sonography was preferred. Otherwise, mammographic imaging was utilized for needle localization.

For ultrasound-guided needle localization, the area of interest was rescanned to confirm the findings. Repeat views were reviewed by the radiologist along with the prior mammographic or sonographic views. Utilizing dynamic sonographic observation, a Homer Mammalok needle (a needle containing the wire to be placed) was inserted. The needle tip was used to traverse through the axis of the lesion with the anchoring wire device deployed at the immediate far side of the lesion.

For small lesions (i.e., typically 4 mm. or less), the needle was placed immediately posterior (towards the patient’s chest wall) relative to the breast lesion. Typically a mammogram was obtained after successful localization to confirm that the sonographic finding is the same as the mammographic finding, and to serve as an “overview” for the surgical team in surgical planning.

For mammographic-guided needle localization, mammographic images were reviewed to determine which projection provides the best visualization and the shortest distance from the skin to lesion. Generally, the cranial to caudal or lateral to medial view was preferred. The breast was then placed onto the appropriate open window of the mammographic compression paddle. If the lesion was within the open window segment of the paddle, Cartesian coordinates on the margin of the window were utilized to localize the skin directly overlying the lesion. The Homer Mammalok needle device was then inserted into the breast at the determined Cartesian coordinates.

All procedures were performed under local infiltration anesthesia of the skin. Localization wires were taped to the affected breast in order to prevent dislocation. Patients were operated on the same day as the localization procedure. An elliptical incision was made around the localized mass, and the incision was carried down around the mass with the localization needle to remove the entire specimen in question. After the specimen was removed, it was marked with sutures for orientation and sent for radiology. After confirmation from the radiologist that the specimen contained the entire lesion, the wound was then closed with 3-0 Vicryl sutures for the deep layer, then 4-0 subcuticular.

### Statistical analysis

All analyses were completed using R v3.3.2 software (R Foundation, Vienna, Austria) by Institutional Statistical Analysts. The descriptive statistics are presented in this paper as percentages, means, or medians, depending on the variable. Tables 1 and 2 depict summary statistics including counts, percentages, medians and range. Tables 3 and 4 utilize independent samples t-tests and chi-square tests to test for mean differences and the significance of associations between subgroups for each variable.

## RESULTS

The study included female patients older than 18 years with lesions of variable BI-RADS classifications. Procedure and surgery related results are outlined in Table 1. Wire migration as a complication of WGL occurred in only one (0.67%) sample patient. Procedural complications were noted in a total of 13 patients; nine (6.04%) patients developed seroma while postoperative hematoma occurred in three (2.01%) patients. Of the 149 sample patients that underwent WGL, 51 (34.22%) patients were identified to have malignancy per the pathology report. Of those 51 patients, 40 (78.43%) had negative margins, with the other 11 (21.56%) had initially positive margins.

All 11 of the patients with positive margins underwent re-excision with either a re-excision lumpectomy or a mastectomy to obtain negative margins. Of the 40 patients with negative margins, seven (17.5%) patients underwent re-excision with either a re-excision lumpectomy or a mastectomy to obtain closer tumor-free margins.

**Table 1.** Procedure and surgery-related results (N = 149 female patients)

**Variable**	**Response**	**All Patients**
Ultrasound or Mammography	Ultrasound	3 (2.0%)
Localization rate	Mammography	63 (42.2%)
	Both (Mammography/US)	80 (53.6%)
	Unknown	3 (2.0%)
	Ultrasound or mammography	149 (100%)
Presence of malignancy	Benign	98 (65.7%)
	Malignant	51 (34.2%)
Histological type	Non-proliferative lesions	42 (28.1%)
	Proliferative lesions without atypia	40 (26.8%)
	Proliferative lesions with atypia	16 (10.7%)
	DCIS	16 (10.7%)
	Invasive lobular	6 (4.0%)
	Invasive ductal	14 (9.4%)
	Invasive ductal + DCIS	15 (10.0%)
Margins for cancer (51 patients)	Negative (% out of 51 patients)	40 (78.4%)
	Positive (% out of 51 patients)	11 (21.5%)
Complications of wire-guided localization	None	148 (99.3%)
	Wire migration	1 (0.6%)
Complications of procedure	Nothing	137 (91.9%)
	Seroma	9 (6.0%)
	Hematoma	3 (2.0%)
Additional surgery	None	131 (87.9%)
	Re-excisional lumpectomies	6 (4.0%)
	Mastectomy	12 (8.0%)

DCIS = ductal carcinoma in situ

Patients and tumor characteristics are shown in Table 2.

**Table 2.** Tumor Characteristics/Findings of Cancer Patients

**Variable**	**Response**	**All Patients**
Age	N Median (range)	51 67.00 (44, 90)
Tumor size (cm)	N Median (range)	51 1.50 (0.04, 6.7)
Specimen volume (cm^3^)	N Median (range)	51 170.00 (0, 180)
Radiologic sizes (cm)	N Median (range)	51 0.82 (0, 3.1)
Radiologic findings (density, microcalcification, or both) (cm)	N Median (range)	51 3.00 (1, 5)
Pre/Postmenopausal	Premenopausal	4 (7.8%)
	Postmenopausal	47 (92.2%)
Tumor localization method	Mammography	19 (37.3%)
	Ultrasound	31 (60.8%)
	Both	1 (1.9%)
Lymph nodes	Negative	49 (96.1%)
	Positive	2 (3.9%)
Histological type	DCIS	16 (31.4%)
	Invasive lobular	6 (11.8%)
	Invasive ductal	14 (27.5%)
	Invasive ductal + DCIS	15 (29.4%)
Grade	Low grade I	21 (41.2%)
	Intermediate grade II	22 (43.1%)
	High grade III	8 (15.7%)
Estrogen-receptor +/-	Negative	5 (9.8%)
	Positive	46 (90.2%)
Progesterone-receptor +/-	Negative	5 (9.8%)
	Positive	46 (90.2%)
HER2 +/-	Negative	35 (68.6%)
	Positive	16 (31.4%)

DCIS = ductal carcinoma in situ

HER2 = Human Epidermal Growth Factor 2

Pathological features are outlined in Table 3. There was an overall significant association between higher BI-RADS Category patients and the presence of malignant pathologies. (p = 0.001) Specifically, we found that of the patients with BI-RADS 5 lesions, 13.3% had invasive lobular carcinoma compared to 0% of patients with BI-RADS 3 and 3.9% of patients with BI-RADS 4. This trend is even more notable with the percentage of patients with infiltrating ductal carcinoma in situ (DCIS) and BI-RADS Category 5 lesions.

**Table 3.** Pathological features of wire-guided localization

**Variable**	**Hist. Type**	**N** **(%)**	**Missing**	**BI-RADS 3**	**BI-RADS 4**	**BI-RADS 5**	**P value**
	0	42 (28.19%)	3 (15.00%)	6 (50.00%)	32 (31.40%)	1 (6.70%)	**0.001**
	1	40 (26.85%)	6 (30.00%)	2 (16.67%)	32 (31.40%)	0 (0.00%)	
	2	16 (10.74%)	4 (20.00%)	2 (16.67%)	9 (8.80%)	1 (6.70%)	
	3	16 (10.74%)	3 (15.00%)	2 (16.67%)	10 (9.80%)	1 (6.70%)	
	4	6 (4.03%)	0 (0.00%)	0 (0.00%)	4 (3.90%)	2 (13.30%)	
	5	14 (9.40%)	2 (10.00%)	0 (0.00%)	6 (5.90%)	6 (40.00%)	
	6	15 (10.07%)	2 (10.00%)	0 (0.00%)	9 (8.80%)	4 (26.70%)	

BI-RADS, Breast Imaging Reporting and Data System

Hist. Type = Histologic Type: 0, non-proliferative lesions; 1, proliferative lesions without atypia; 2, proliferative lesions with atypia; 3, ductal carcinoma in situ; 4, invasive lobular; 5, invasive ductal; 6, invasive ductal + ductal carcinoma in situ.

Of the patients with a BI-RADS 5 lesion, 40% were found to have a pathological diagnosis of IDC, compared to 0% of patients with BI-RADS 3 and only 5.9% of patients with BI-RADS 4. Again, of the patients classified as a BI-RADS 5 on imaging, 26.7% had IDC and ductal carcinoma in situ compared to 0% of the patients classified as BI-RADS 3 and 8.8% of the patients with BI-RADS 4. Thus, we can see that there is statistically significant value in the BI-RADS 5 classification and indication for surgical excision of the lesion.

Of the 149 patients, 102 (68.45%) lesions were categorized as BI-RADS 4. Of these, 37 (36.27%) were subcategorized into 4A, 4B or 4C. There were 29 (28.43%) patients found to have cancer in BI-RADS category 4, with 19 (65.51%) of them being subcategorized. The subcategorizations of 4A, 4B and 4C with positive lesions were two (6.89%), three (10.34%) and five (17.24%), respectively. The non-significance of histologic type differences are depicted in Table 4, likely due to subcategory frequencies. (p = 0.229) The only interesting finding between the variables and surgical margins was an association between invasive lobular carcinoma and positive margins: 50% of invasive lobular carcinoma had positive margins. This likely reflects the infiltrative process of lobular carcinoma and could be related to the difficulty in visualizing the extent of the tumor with mammography and/or ultrasound.

**Table 4.** Characteristics of surgical margin in the wire-guide localization group

**Variable**	**Response**	**Overall**	**Surgical Margin Negative**	**Surgical Margin Positive**	**p-value**
**Age**	N Median (range)	51 67 (44, 90)	40 68.50 (44, 90)	11 62.00 (44, 80)	0.199
**Pre/Postmenopausal**	Premenopausal	4 (7.84%)	2 (5.00%)	2 (18.18%)	0.420
	Postmenopausal	47 (92.16%)	38 (95.00%)	9 (81.82%)	
**Tumor size (cm)**	N Median (range)	51 1.5 (0.04, 60.75)	40 1.26 (0.04, 60.75)	11 3.46 (0.08, 12.4)	0.784
**Specimen volume** **(cm^3^)**	N Median (range)	51 170 (0, 1800)	40 216.50 (0, 864)	11 133.00 (3, 1800)	0.887
**Lymph nodes**	Negative	49 (96.08%)	39 (76.47%)	10 (19.61%)	0.607
	Positive	2 (3.92%)	1 (2.50%)	1 (9.09%)	
**Histological type**	DCIS	16 (31.37%)	14 (35.00%)	2 (18.18%)	0.229
	Invasive lobular	6 (11.76%)	3 (7.50%)	3 (27.27%)	
	Invasive ductal	14 (27.45%)	12 (30.00%)	2 (18.18%)	
	Invasive ductal + DCIS	15 (29.41%)	11 (27.50%)	4 (36.36%)	
**Grade**	Low grade I	21 (41.18%)	17 (42.50%)	4 (36.36%)	0.641
	Intermediate grade II	22 (43.14%)	16 (40.00%)	6 (54.55%)	
	High grade III	8 (15.69%)	7 (17.50%)	1 (9.09%)	

DCIS = ductal carcinoma in situ

## DISCUSSION

It has been demonstrated that early stage diagnosis of clinically non-palpable malignant lesions can be achieved by utilizing many imaging modalities such as mammography, ultrasonography and image-guided sampling for histopathology.^13^ In conservative breast surgery, the biggest challenge for the surgeon remains obtaining clear surgical margins. Positive excision margins significantly increase local recurrence,^14^ because re-excisional lumpectomy or mastectomy must then be performed.^15^

For more conservative breast lesion localization, the use of WGL metallic markers,^2^ ultrasound guided surgery,^16^ and the radio-guided occult lesion localization technique^17^ are now routinely used. Wire-guided biopsy is a well-known technique that has been used since the 1970s. In our study, WGL was successful in 100% of benign sample patients, with overall similar fewer complications related to surgical technique.

In our study, positive surgical margins were found in 11 (21.6%) of our 51 malignant lesions. Of the 11 patients with positive margins, seven (63.6%) underwent mastectomies, with the other four (36.4%) undergoing re-excisional lumpectomies. The total number of mastectomies performed as re-excisions in this study was 12, this number including the seven previously listed, and the five mastectomies that were performed after initial negative margins.

The total number of mastectomies needed to achieve satisfactory margins in patients after initial WGL was 10 (19.6%), as compared to the 20% reported in the literature.^18^ There were two additional patients who underwent re-excisional lumpectomies after initial negative margins to obtain more satisfactory negative margins.

The BI-RADS is a standardized radiological reporting system that was established in 1993 by the American College of Radiology and has been modified multiple times to include other radiological modalities (e g., ultrasonography) that was initially oriented to mammography.^19^ Over the course of the BI-RADS creation and validation, several meta-analyses, retrospective and prospective studies were performed showing a moderate variability in the reviewed literature among positive predictive values (PPV) of BI-RADS categories 3, 4 and 5, revealing PPVs ranging respectively between 0.8% and 9%, 16.8% and 34%, and 81% and 95%.^20–22^

Our study found a very statistically significant association between BI-RADS and histology type. Notably, BI-RADS 5 category had a clear correlation with malignancy, showing that 40% had IDC compared to 0% of patients with BI-RADS 3 and 5.9% of patients with BI-RADS 4. When examining the PPV of BI-RADS 5 patients in Table 2, we demonstrated a PPV of 86.7% (13 true positives out of a total of 15 initial “positives”). The ranges of the PPV of categories 3 and 4 were 16.7% and 28.4%, respectively.

Our category 3 lesions PPV of 16.7% was higher than prior literature of less than 2%,^23^ perhaps due to the types of patients receiving care in this particular community-based setting and/or the independent radiologist’s interpretations. However, our overall findings from BI-RADS Category 3 through 5 generally match earlier published rates, justifying the optional use of needle and/or surgical breast biopsy at the discretion of the treating physician.

An issue can arise when considering the most appropriate treatment of BI-RADS Category 4 cases. BI-RADS 4 cases are often considered suspicious for malignancy on imaging, most commonly assigned a category between 3 through 5 approximately 70% of the time.^24^ With a PPV of 28.4% shown in this study, we conclude that the biopsies of 71.6% of our BI-RADS sample WGL biopsy specimens biopsied will come back benign. This suggests to us that there remains a high amount of unneeded procedural excisions being performed for benign breast disease.

In 2003, the American College of Radiology produced the fourth edition of the Breast Imaging Reporting and Data System. This edition subcategorized BI-RADS 4 into 4A, 4B and 4C to stratify the wide range of lesions that needed interventional procedures. The subcategorization identifies 4A, 4B and 4C as needing intervention but with low suspicion for malignancy, lesions with intermediate suspicion, and moderate suspicion but not classic for malignancy, respectively. With this, the chance for malignancy for 4A, 4B and 4C are 2%-9%, 10%-49% and 50%-94%, respectively.^25^

In discussion with our radiology department, we learned that there has been no formal healthcare system or national protocol outlining specific details on how to determine when to use the BI-RADS 4 subcategorization. The decision whether to categorize a lesion as category 4 or subcategorize into 4A, 4B or 4C is still solely left up to the reviewing radiologist, allowing them to analyze the data and determine what classification the lesion best resides within.

When further examining our data in conjunction with the newer subcategorization of BI-RADS Category 4, 102 (68.45%) total lesions were categorized as BI-RADS 4. In this subgroup, 37 (36.27%) were subcategorized into 4A, 4B or 4C. There were 29 (28.43%) patients found to have cancer in the BI-RADS category 4, with 19 (65.51%) of them being subcategorized. This correlates with the earlier results of one project that demonstrated that the subcategorizations have a higher PPV than the original Category 4 grouping.^25^ The subcategorizations of 4A, 4B and 4C with positive lesions were two (6.89%), three (10.34%) and five (17.24%), respectively.

After these subgroup analyses, we have concluded that a more formal national BI-RADS protocol still needs to be established, assigning more lesions to the subcategorizations of 4A, 4B and 4C instead of simply assigning a category 4 classification. In discussion with radiologists at our hospital, we have also concluded that our trying to improve our PPV without development of a more empirically-based protocol might come at the expense of lowering the overall breast cancer detection rate.

By further delineating BI-RADS 4 cases by radiologic findings and comparing them to pathology results, radiologists could more accurately determine subsets of BI-RADS 4, resulting in less unnecessary procedures. Conclusions drawn from future works are needed to update protocol guidelines with newly defined 4A, 4B and 4C subcategorizations that can clarify the distinguishing characteristics of BI-RADS 4 cases to avert unnecessary benign biopsies.

### Limitations

The analytic sample for this study came from a community-based imaging department in one Michigan site. As depicted in Table 3, there were also 20 apparently eligible patients who we were unable to find BI-RADS classification imaging data in their electronic health records since their imaging was done at outside facilities.

### Conclusions

Our study results should raise concern that too many patients undergoing breast cancer workup are likely undergoing unnecessary procedures. We would suggest that radiologists and surgeons across the nation become more familiar with the 5^th^ edition BI-RADS 4 diagnostic subcategorizations. Future retrospective studies to help identify the distinguishing characteristics of benign vs malignant lesions in BI-RADS 4 are greatly needed. Once better delineated from larger samples, these characteristics could be used to better define BI-RADS subcategorizations of 4A, 4B and 4C and ultimately lead to fewer unnecessary breast excisional surgeries.

### Conflict of Interest

The authors declare no conflict of interest.
